# Mortality after low trauma hip fracture: a prospective cohort study

**DOI:** 10.1186/1471-2474-13-143

**Published:** 2012-08-10

**Authors:** Majid Valizadeh, Saeideh Mazloomzadeh, Somayeh Golmohammadi, Bagher Larijani

**Affiliations:** 1 Metabolic disease research center, Zanjan University of Medical Sciences, Zanjan, Iran; 2 Faculty of Medicine, Zanjan University of Medical Sciences, Zanjan, Iran; 3 Endocrinology & Metabolism Research Center (EMRC), Tehran University of Medical Sciences, Tehran, Iran

## Abstract

**Background:**

Various risk of mortality due to hip fracture has been reported by different studies. There is scarce controlled study on hip fracture mortality from developing countries and no data from Middle East region. The objective of this study is to determine mortality and its risk factors one year after low trauma hip fracture.

**Methods:**

One hundred and two patients after hip fracture not caused by high impact injuries or local bone diseases followed up prospectively for one year. Control group consisted of sex and age matched patients admitted to ophthalmology ward for eye surgery. Data about comorbidity obtained from both groups at baseline. Functional state and health-related quality of life for the participants were measured using RDRS-2 and SF-36 questionnaires, respectively.

**Results:**

The overall survival was 83% in cases and 92% in controls (log rank test 3.62, df = 1, P = 0.057). Early mortality within the first 6 months of observation was significantly higher in patients than controls (13 in patients vs. 2 in controls) (log rank test 8.84, df = 1, P = 0.003). The risk of mortality in the first year after fracture was significantly and independently associated with age and baseline RDRS score. By the end of follow-up, in the patient group, 55.4% of survivors were able to walk without any assistance and 10.8% were not able to walk.

**Conclusions:**

The risk of mortality within the first 6 months of observation was significantly and independently associated with low trauma hip fracture. However, age and baseline RDRS score were independent predictors of mortality in the first year following hip fracture.

## Background

Hip fracture is the most serious complication of osteoporosis. Studies have shown that mortality increases significantly after hip fracture and disability is high subsequent to its occurrence [1,2]. The range of mortality risks up to one year after the fracture were reported from 5% to 50% in various studies [2-12]. Also factors associated with mortality is different from one study to other one such as age, gender, presence of comorbid diseases, prefracture functional ability and post fracture complications [8,13-15]. Most epidemiologic data on hip fracture and its consequences originate from western countries (Scandinavian countries and North America) with higher prevalence of this type of fracture. There are few data from developing regions of the world and no prospective controlled study about hip fracture outcome and its associated factors from Middle East. The aim of this study was to determine mortality and morbidity and its risk factors after one year of follow up for patients with hip fracture in comparison with control group using a prospective design.

## Methods

This was a prospective cohort study in which the exposure was presence of hip fracture and the outcome was mortality. Case group comprised of 102 consecutive eligible patients aged ≥ 50 who admitted at Zanjan Mousavi hospital with a low trauma hip fracture that was not related to local bone disease or metastasis. Mousavi hospital is the major referral center for trauma in Zanjan province. Low trauma was defined as falls from standing height or less. 41(40.2%) fractures affected the femoral neck, and 61 (59.8%) were intertrochanteric. The fractures of femoral neck was operated using Open Reduction Internal Fixation (ORIF) with 3 parallel screws or hemiarthroplasty methods according to the age of patients and Dynamic Hip Screw (DHS) was used to treat those with Intertrocantheric fractures.

Control group consisted of sex and age matched (± 5 years) patients admitted to ophthalmology ward for eye surgery due to any cause without history of hip fracture. The majority of patients in the ophthalmologic ward are at the same age as patients with hip fracture.

Data were collected on age, residence (own home, Relative home), concomitant diseases (Dementia, Parkinson, CVA, IHD or CCU admission, Hypertension, Dyslipidemia, Diabetes, Rheumatoid arthritis and osteoarthritis) and drugs at baseline. BMI was calculated for 62 fracture cases and 100 controls whose data on height and weight was available. Missing observations were due to the inability of patients in standing on the scale for weight measurement. Functional state and health-related quality of life for the participants were measured by a trained interviewer using RDRS-2 and SF-36 questionnaires that filled both at study entry and the end of follow up. Functional status of hip fracture patients was asked from themselves or their families based on their prefracture condition. The RDRS-2 questionnaire comprises 18 items grouped into three domains: activities of daily living (eight items, including eating, walking, mobility, bathing, dressing, toileting, grooming, and adaptive tasks), degree of dependence (seven items, including communication, hearing, sight, diet, stay in bed during the day, incontinence, and medication), and cognitive impairment (three items, including mental confusion, uncooperativeness, and depression). These items are ranked on a 4-point scale, with 0 indicating the best function and 3 the worst. Therefore, functional status of patient deteriorates with increasing the score of RDRS-2. Vital status of the subjects was evaluated at 1, 3, 6, 12 months by contact via phone with the patients or their families. The study was approved by the Ethical Committee of the Zanjan University of Medical Sciences and informed consent has been obtained from all subjects.

The Kolmogorov-Smirnov test was used to evaluate the distribution of quantitative variables. Values were expressed as number (percentage), and mean ± standard deviation, as appropriate. Comparisons were performed by chi-square test for categorical variables, independent or paired *T*-test for normally distributed, and Mann–Whitney or Wilcoxon test for non-normally distributed.

The Kaplan-Meier method was used to estimate the probability of survival at follow-up. Comparisons were performed using the log-rank test. A Cox’s proportional hazards model was constructed to examine the association between mortality and the relevant variables. All statistical analyses were performed using the SPSS PC version 16.0 computer software program for Windows (SPSS, Chicago, IL, USA).

## Results

Baseline characteristics of cases and controls are demonstrated in Table 1. BMI was identified in 62 cases and 100 controls. The mean of BMI was lower in the cases compared to controls (P = 0.03). No significant differences were observed between two groups for age, gender, residence and number of comorbidities.

**Table 1:** **Characteristics of patient and control groups**^**a**^

**Characteristics**	**Cases (n = 102)**	**Controls (n = 101)**	**P-value**
Age	73.7 ± 8.7	72.4 ± 9.3	0.3
BMI	23.3 ± 4.2	24.9 ± 4.5	0.03
Gender			
Male	47 (46.1)	49 (48.5)	0.73
Female	55 (53.9)	52 (51.5)	
Residence			0.14
Own home	95 (94.1)	89 (88.1)	
Relatives home	6 (5.9)	12 (11.9)	
Comorbidities			
0	27 (26.5)	23 (22.8)	0.65
1-2	55 (53.9)	61 (60.4)	
≥ 3	20 (19.6)	17 (16.8)	

^a^Results expressed as mean ± standard deviation, and number (percentage) as appropriate.

### Mortality

**Figure 1:**
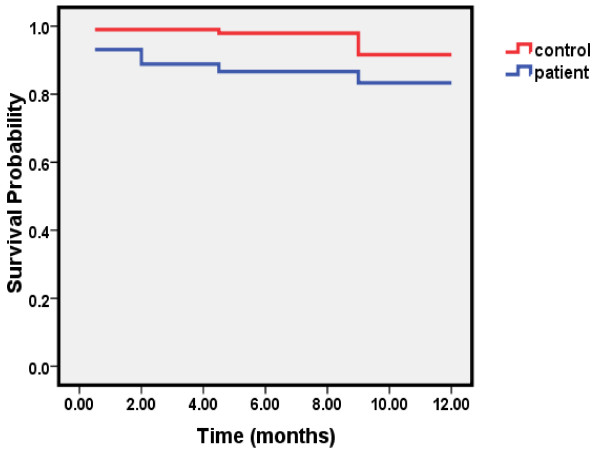
Survival among the case and control groups.

The mean time of follow-up was 11.3 ± 2.5 (range, 1–12 months). At the end of follow-up, 16 patients with fracture died compared to 8 in controls. The Kaplan-Meier survival curve indicates a greater number of deaths in patients with fracture than controls (Figure 1). The overall survival was 83% in cases and 92% in controls (log rank test 3.62, df = 1, P = 0.057). Early mortality or the number of deaths within the first 6 months of observation was significantly higher in patients than controls (13 in patients vs. 2 in controls) (log rank test 8.84, df = 1, P = 0.003). However, the number of deaths within the second 6 months of follow-up was 3 in the patients and 6 in the controls (log rank test 0.57, df = 1, P = 0.45).

The risk of mortality in the first year following hip fracture was significantly associated with age, BMI and baseline RDRS score using univariate Cox regression analysis (Table 2). The hazards ratio for baseline RDRS score remained significant after adjusting for age. In the multivariate analysis, using age, gender, the presence of hip fracture, and baseline RDRS score in the model, the risk of mortality within six months was significantly and independently higher in patients than controls (HR: 6.26, 95%CI: 1.40-28.02). However, the risk of mortality in the first year following hip fracture was not significantly elevated (HR: 2.07, 95%CI: 0.87-4.91). Age and baseline RDRS score were significant and independent predictors of mortality in the first year following hip fracture (Table 3).

**Table 2:** **Hazard ratio and 95**% **confidence intervals of mortality in the first year following hip fracture by study variables**

**Characteristics**	**HR (95**% **CI)**	**P-value**	**HR (95**% **CI)**^**a**^	**P-value**
Hip fracture				
No	1.00	0.07	1.00	0.08
Yes	2.20 (0.94-5.15)		2.17 (0.93-5.07)	
Age (for each year increase in age)	1.08 (1.02-1.14)	0.009	-	
Age (years)			-	
≤ 70	1.00	0.02		
> 70	4.14 (1.24- 13.89)			
BMI (for each unit increase in BMI)	0.82 (0.69-0.98)	0.03	0.84 (0.69-1.01)	0.07
Gender				
Female	1.00	0.13	1.00	0.29
Male	1.90 (0.83-4.34)		1.58 (0.68- 3.64)	
Residence				
Own home	1.00	0.49	1.00	0.92
Relatives home	1.53 (0.46-5.16)		1.06 (0.31-3.65)	
Comorbidities				
0	1.00	0.28	1.00	0.25
1-2	0.51 (0.20-1.30)		0.53 (0.21-1.35)	
≥ 3	0.98 (0.34-2.82)		1.15 (0.40-3.33)	
Baseline RDRS score	1.06 (1.02-1.11)	0.003	1.05 (1.01-1.09)	0.028

^a^Age adjusted.

**Table 3:** Hazard ratio and 95% confidence intervals of mortality within the first 6 months and in the first year following hip fracture by study variables in multivariate analysis

**Characteristics**	**Mortality within six months**		**Mortality in the first year**	
	**HR (95% CI)**	**P-value**	**HR (95% CI)**	**P-value**
Hip fracture				
No	1.00	0.017	1.00	0.098
Yes	6.26 (1.40-28.02)		2.07 (0.87-4.91)	
Age (for each year increase in age)	1.05 (0.98-1.13)	0.17	1.06 (1.004-1.13)	0.037
Gender				
Female	1.00	0.33	1.00	0.16
Male	1.68 (0.59-4.77)		1.81 (0.79-4.18)	
Baseline RDRS score	1.04 (0.99-1.09)	0.13	1.05 (1.004-1.09)	0.029

### Morbidity

**Figure 2:**
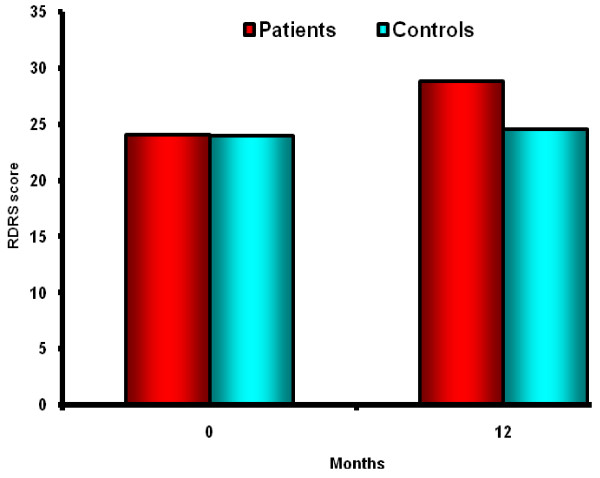
RDRS at Base line and at the end of follow up in case and control group.

RDRS Questionnaire scores were available for all cases and controls at baseline and for 74 patients with fracture and 87 controls at the end of follow-up. The mean RDRS score in the hip fracture patients was 24.1 ± 6.4 at baseline and 28.8 ± 9.4 at 12 months (P < 0.0001) and in control group, the mean score was 24.0 ± 4.6 at baseline and 24.6 ± 5.6 at 12 months (P = 0.71) (Figure 2).

By the end of follow-up, in the patient group, 55.4% were able to walk without any assistance and 10.8% were not able to walk. However, in the control group, these proportions were 93.1% and 1.1%, respectively (P < 0.0001).

## Discussion

The present study revealed that risk of one year mortality in low trauma hip fracture group is twice greater than controls. However, the risk was not statistically significant. Most deaths in the case group occurred in the first 3 months after fracture (68%), however, deaths in the control group were more frequent in the second 6 month of the year (75%). Numerous studies have reported that mortality increased after hip fracture but the risk varied from one study to another according to age, sex and other characteristics of population under study [6,12,14-25]. The risk of first year mortality reported by Vestergaard et al. [15] and Roche et al. [26] were 29% and 33%, respectively, which are both higher than that of our study (15.6%). Higher mean age and serious comorbidity such as cancer at baseline in their patients may explain some of these differences. The mean age of our patients at the time of fracture (73.7 ± 8.7) was smaller than many other studies reported from western countries. With respect to age, which is one of the most important risk factor for mortality, perhaps this subject was responsible for less mortality after operation in our study.

We found that the Hazard ratio of early mortality (during the first 6 months after fracture) was significantly 6.26 times greater in fracture cases than controls. Higher mortality during the first several months after fracture was also reported in other studies [13,15,24]. Later mortality was varied since some studies indicated that excess mortality continues after this time period but others reported no excess mortality after that [7,14,24,27].

Since there was no significant difference for the number of co-morbidities and SF 36 scores (data not reported), both indicators of general health, between the case and control groups, the high early mortality observed in our study, can be perceived that hip fracture is the primary cause of death shortly after the event. This is in accordance to the study by Vestergaard et al. [15], however, we did not investigate causes of death in our study and RDRS was 2 scores worse in cases that is not clinically important.

We found age, BMI and pre-fracture RSDS scores as prognostic factors for mortality, in the univariate analysis. The risk of death increased with advancing age and became more prominent after 70 years as indicated in Table 2. Several studies have identified mortality rates following hip fracture increases with advancing age [7,11,19,21,28]. However, three studies have indicated that younger age at fracture lead to excess mortality compared to the controls and this risk decreases with advancing age [11,14,17,23]. In our study, age was an independent risk factor for mortality in the first year following hip fracture, but not for early mortality (within six months), after controlling the effect of other variables in the multivariate analysis.

In this study, a higher proportion of hip fracture was indicated in males compared to females. In our previous study, the female to male ratio of hip fracture for Iranian habitants in Zanjan province was 1.0 or less in almost all age-groups which is lower than that was reported in most western countries but it is similar to the low incidence rate of hip fracture areas in Asia such as rural areas in Turkey and Beijing, China [29,30]. The complete etiology of HF in this area is not well understood. The main reason for this difference is related to lower incidence in women as the statistics shows, but the rate of hip fracture in Iranian men resembles recorded rate in most western countries. In this regards the present observe confirms the results of previous study on the epidemiology of hip fracture in Iran by Moayyeri et al. [31]. These results are not in consistent with low bone density demonstrated in Iranian women. It is suggested that the incidence of fall injuries in men is higher than women in the under 60 age group. In the above 60 age group, the risk of incidentally falling elevates dramatically in women and it is higher than men. Interestingly, results of many suggest that breast feeding may reduce the risk of hip fractures in women in elderly. In our province breastfeeding is common and this may explain the observed low HF incidence in the area.

Tosteson and co-workers [24], reported that in addition to age, BMI, and pre-fracture health status, sex is also a prognostic factor of mortality after hip fracture but in our study sex did not influence mortality. Similar to the findings of Tosteson, many researchers found that males had worse outcomes than females following hip fracture [8,32-34]. On the other hand, a few other earlier studies concluded that gender is not an independent predictor of mortality in multivariate analysis [2,35]. In our analysis presence and the number of co-morbidity did not have an effect on the one year survival and this finding is in agreement with the results of the study conducted by Cipitria et al. [36]. In contrast, the majority of studies indicated that co-morbidities increases the risk of mortality [14,15,19,21,23,24]. Baseline RDRS scores that directly indicates functional ability influenced mortality in our cases, with a HR of 1.06. Our analysis has also indicated that an increase in BMI decreases mortality, an effect that is reported only in two of the previous studies, however, missing data in our case group for BMI was about 40% [14,24].

More than half (55%) of survivors in case group of our study, were able to walk without any assistance, 34% could walk with crutch or walker and 11% were bedridden. Pande et al. [8] reported that only 36% of their patients (males) could walk independently 12 months after fracture and 15% could not walk. The proportion of those who became bedridden was 9% in a study by Wong and colleagues [37], which is close to our findings. The mean age and RDRS score of patients were statistically different according to the ability to walk after operation (69.8 and 22.5 for those who were able to walk without any assistance, 76.1 and 25.0 for those who were able to walk with assistance and 73.1 and 29.8 for those who were not able to walk, P = 0.01 and P = 0.009, respectively).

Our study has some strength; that was a prospective cohort with sex and age matched control group and also the dropout rate was less than 10% in both groups. However, data related to health and functional ability before fracture was gathered retrospectively after admission due to fracture and may be affected by the memory of the patients (recall bias).

## Conclusion

The risk of mortality within the first 6 months of observation was significantly and independently associated with low trauma hip fracture. However, age and baseline RDRS score were independent predictors of mortality in the first year following hip fracture. At the end of follow-up, nearly to half of patients with fracture were not able to walk without any assistance.

## Competing interests

The authors declare that they have no competing interests.

## Authors’ contributions

MV contributed to design, acquisition of data, interpretation of data and drafting and revising the manuscript. SM contributed to design, performing statistical analysis and interpretation of data and drafting the manuscript. SG contributed to design and acquisition of data. BL contributed to design and interpretation of data. All authors read and approved the final manuscript.

## Financial support

This study was financially supported by the Zanjan University of Medical Sciences and Endocrinology & Metabolism Research Center (EMRC), Tehran University of Medical Sciences, Tehran, Iran.

## Pre-publication history

The pre-publication history for this paper can be accessed here:

http://www.biomedcentral.com/1471-2474/13/143/prepub
